# Voltage-Gated Potassium Channel Kv1.3 Is Highly Expressed in Human Osteosarcoma and Promotes Osteosarcoma Growth

**DOI:** 10.3390/ijms140919245

**Published:** 2013-09-23

**Authors:** Jin Wu, Daixing Zhong, Xinyu Wu, Mo Sha, Liangqi Kang, Zhenqi Ding

**Affiliations:** 1 Department of Orthopaedics, the Affiliated Southeast Hospital of Xiamen University, Zhangzhou 363000, China; E-Mails: wuxinyu0102@163.com (J.W.); quangao175th@163.com (M.S.); 2 Department of Thoracic Surgery, the Affiliated Tangdu Hospital of Fourth Military Medical University, Xi’an 710038, China; E-Mail: xingxing.1230@hotmail.com; 3 Department of Neurology, the Affiliated Southeast Hospital of Xiamen University, Zhangzhou 363000, China; E-Mail: yuxin0506@163.com

**Keywords:** Kv1.3, osteosarcoma, shRNA, cell proliferation, apoptosis, immunohistochemistry

## Abstract

Deregulation of voltage-gated potassium channel subunit Kv1.3 has been reported in many tumors. Kv1.3 promotes tumorigenesis by enhancing cell proliferation while suppressing apoptosis. However, the expression and function of Kv1.3 in osteosarcoma are unknown. In the present study, we detected the expression of Kv1.3 in human osteosarcoma cells and tissues by RT-PCR, Western blot and immunohistochemistry. We further examined cell proliferation and apoptosis in osteosarcoma MG-63 cells and xenografts following knockdown of Kv1.3 by short hairpin RNA (shRNA). We found that Kv1.3 was upregulated in human osteosarcoma. Knockdown of Kv1.3 significantly suppressed cell proliferation and increased apoptosis as demonstrated by enhanced cleavage of poly (ADP-ribose) polymerase (PARP) and the activation of Caspase-3/7. Furthermore, adenovirus delivered shRNA targeting Kv1.3 significantly inhibited the growth of MG-63 xenografts. Taken together, our results suggest that Kv1.3 is a novel molecular target for osterosarcoma therapy.

## 1. Introduction

Osteosarcoma is the most common malignant primary bone tumor, particularly among children and adolescent [1,2]. Osteosarcoma has high local aggressiveness with a metastasis tendency most commonly to the lung, pleura, bone, and heart [3]. Consequently, long-term survival is dismal as most osteosarcoma patients often die of systemic metastasis, especially diffuse lung metastases [4,5]. Despite recent advances in aggressive treatment modalities, such as adjuvant chemotherapy and wide tumor resection, the five-year survival rate of osteosarcoma patients is only 55%–60%, and lung and skeletal metastases occurs in approximately 30% to 40% of these patients [6]. Meanwhile, high-dose chemotherapy has a number of adverse effects such as gastrointestinal reactions, bone marrow suppression, cardiac toxicity, and renal toxicity [7]. Thus, it is urgent to develop new treatments for osteosarcoma.

Growing evidence has shown that voltage-gated potassium channels (Kv channels) play an important role in the development and growth of cancers [8–10]. Among Kv channels, potassium voltage-gated channel, shaker-related subfamily, member 3, also known as KCNA3 or Kv1.3, is a protein encoded by the KCNA3 gene, which appears to be intronless and is clustered together with KCNA2 and KCNA10 genes on chromosome 1 [11]. Kv1.3 regulates membrane potential and Ca^2+^ signaling in human T cells and thus plays an important role in T cell proliferation and activation [12]. In addition, Kv1.3 is known to be expressed in the inner mitochondrial membrane of lymphocytes, and apoptotic protein Bax could insert into the outer mitochondrial membrane and occlude the pore of Kv1.3 via a lysine residue [13]. Therefore, Kv1.3 modulation appears as one key mechanism that contributes to apoptosis [14,15]. Interestingly, recent studies have shown that Kv1.3 is implicated in the growth and proliferation of various cancer cells, such as breast, gastric, gliomas, and prostate cancer [16–19]. The tight relationship between Kv1.3 expression and cancer indicates that Kv1.3 is a molecular target for the development of anticancer therapies [20]. Nevertheless, there is currently debate regarding the relationship between the aberrant Kv1.3 expression and malignancy and invasiveness of cancers. For example, there are apparently controversial conclusions regarding the correlation between Kv1.3 level and tumorigenicity in breast cancer, gliomas, and prostate cancer [16,18,19]. Therefore, further investigations are needed to elucidate the function of Kv1.3 in the tumorigenesis of different cancers.

Up to now, little is known about the relationship between the expression status of Kv1.3 and human osteosarcoma. Moreover, the biological functions of Kv1.3, during the multiple step processes of tumorigenesis of osteosarcoma, are largely unclear. To address these questions, we examined the expression of Kv1.3 in human osteosarcoma by RT-PCR, Western-blot and immunohistochemistry, and determined cell proliferation and apoptosis following knockdown of Kv1.3 by RNA interference (RNAi) in cultured osteosarcoma MG-63 cells. We further monitored tumor growth of osteosarcoma MG-63 mouse xenografts after downregulation of Kv1.3 by adenovirus delivered shRNA.

## 2. Results

### 2.1. Aberrant Expression of Kv1.3 in Human Osteosarcoma

**Figure 1:**
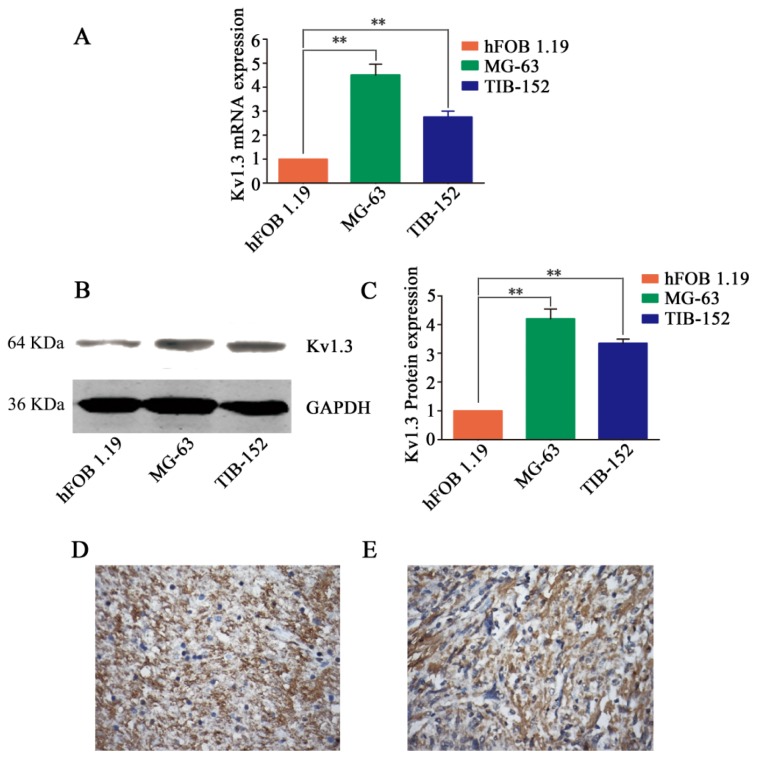
Kv1.3 is highly expressed in osteosarcoma cells and tissues. (**A**) RT-PCR analysis of Kv1.3 mRNA level in hFOB 1.19, MG-63, and TIB-152 cells. Kv1.3 mRNA level in hFOB 1.19 cells was set as 1. β-actin was used as the internal standard; (**B**) Western blot analysis of Kv1.3 protein expression in hFOB 1.19, MG-63, and TIB-152 cells; (**C**) Densitometry analysis of Kv1.3 protein level. GAPDH was loading control. Kv1.3 protein level in hFOB 1.19 cells was set as 1 and the results were expressed as mean ± SD (*n =* 3). ** *p* < 0.01; (**D**) Immunohistochemical staining of Kv1.3 in a human brain specimen (positive control); and (**E**) Immunohistochemical staining of Kv1.3 in human osteosarcoma specimens. Images were captured using an OLYMPUS light microscope equipped with a CCD color camera at 400× magnification.

We first compared the mRNA and protein expression levels of Kv1.3 in human osteosarcoma cell line MG-63, human Jurkat cell line TIB-152 (positive control [18]) and osteoblastic cell line hFOB 1.19. The mRNA and protein levels of Kv1.3 in MG-63 cells were significantly increased to a level approximately 4.5 times (Figure 1A) and 4.2 times (Figure 1B,C), respectively higher than that in hFOB 1.19 cells. Next, we detected the expression of Kv1.3 in human osteosarcoma tissues and found that positive Kv1.3 staining was detected in 29/41 (70.7%) osteosarcoma. As positive control, Kv1.3 staining was detected in normal human brain samples (Figure 1D). A representative staining of Kv1.3 in human osteosarcoma tissues was shown in Figure 1E. These results demonstrated that Kv1.3 was highly expressed in human osteosarcoma cell lines and tissues.

### 2.2. Kv1.3 Knockdown Inhibits MG-63 Cell Proliferation *In Vitro*

**Figure 2:**
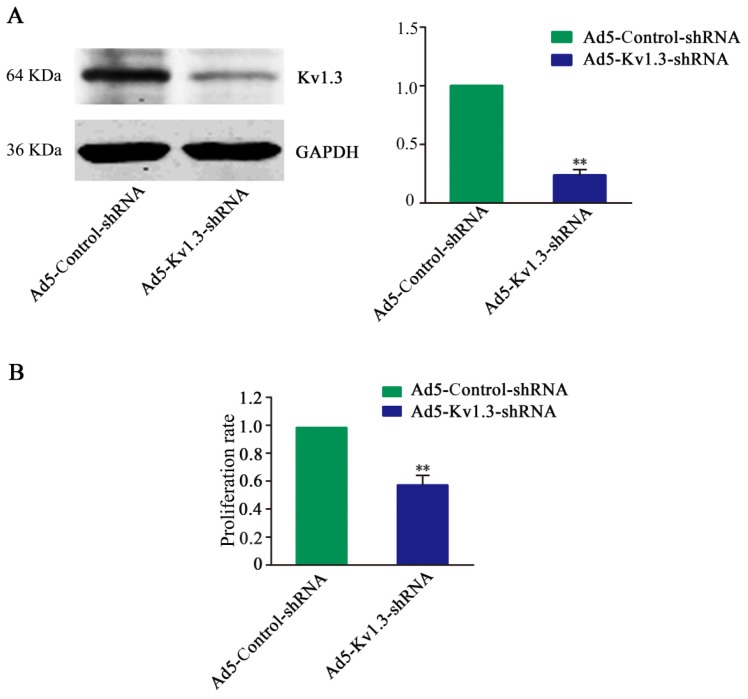
Kv1.3 knockdown leads to reduced proliferation of MG-63 cells *in vitro*. (**A**) The knockdown efficiency of Kv1.3-shRNA in MG-63 cells was examined by Western blot analysis. GAPDH was loading control; (**B**) CCK-8 assay showing the proliferation of MG-63 cells after infection by Kv1.3-shRNA. Data were normalized to Control-shRNA and presented as mean ± SD (*n =* 3). ** *p* < 0.01 *vs.* Ad5-Control-shRNA group.

To explore the potential function of Kv1.3 in the tumorigenesis of human osteosarcoma, we first knocked down Kv1.3 in cultured MG-63 cells by infecting Ad5-Kv1.3-shRNA. Compared to Ad5-Control-shRNA infected cells, Ad5-Kv1.3-shRNA infected MG-63 cells displayed apparent reduction of Kv1.3 protein (Figure 2A), indicating the high knockdown efficiency of Ad5-Kv1.3-shRNA. Following this, we checked the cell proliferation using CCK-8 assay following infection of MG-63 cells with Ad5-Kv1.3-shRNA. As shown in Figure 2B, Ad5-Kv1.3-shRNA inhibited the proliferation of MG-63 cells by 42.8%.

### 2.3. Kv1.3 Knockdown Inhibits Osteosarcoma Growth *In Vivo*

**Figure 3:**
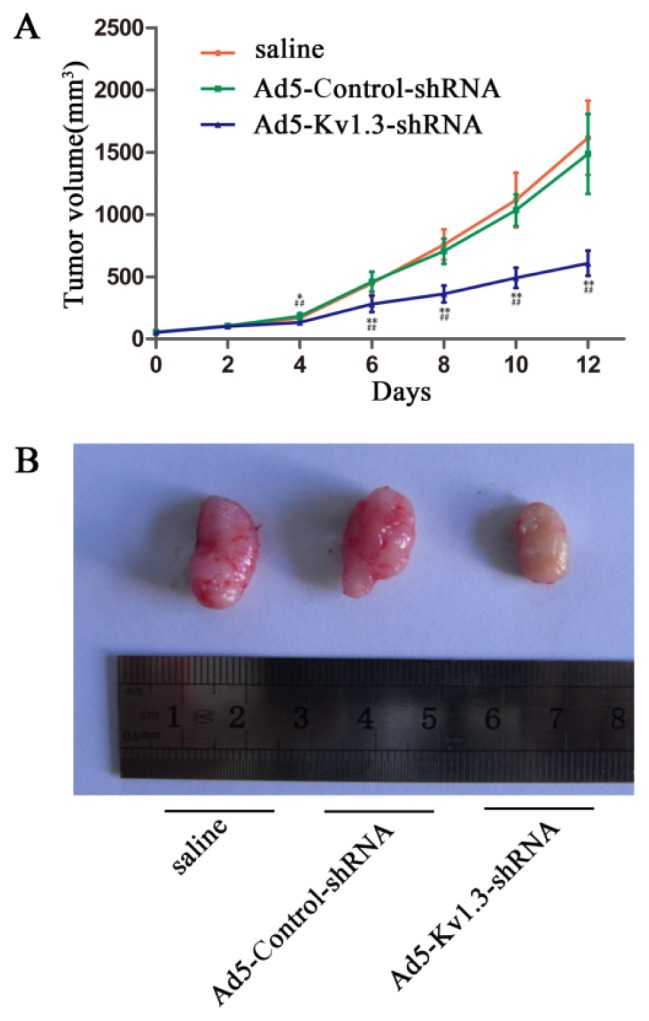
Kv1.3 knockdown inhibits the growth of MG-63 xenografts in nude mice.

To extend our *in vitro* observation on cultured MG-63 cells, we made a xenograft model of osteosarcoma using nude mice, and treated the xenografts by intra-tumor injection of Ad5-Kv1.3-shRNA, Ad5-Control-shRNA, or saline. As shown in Figure 3, the tumor volume in Ad5-Kv1.3-shRNA injected animals was significantly smaller than those in saline or Ad5-Control-shRNA injected animals. These *in vivo* data suggest that Kv1.3 promotes osteosarcoma growth.

### 2.4. Kv1.3 Knockdown Induces Apoptosis of MG-63 Cells

**Figure 4:**
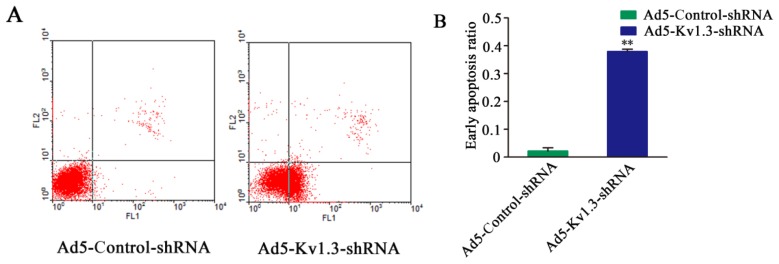
Kv1.3 knockdown induces early apoptosis of MG-63 cells. (**A**) Flow cytometry analysis of Annexin V/PI in MG-63 cells after infection with Ad5-Kv1.3-shRNA. Cells infected with Ad5-Control-shRNA were used as the control. Cells in the right lower quadrant indicated Annexin-positive, early apoptotic cells; (**B**) Significant increase of early apoptotic rate in Ad5-Kv1.3-shRNA infected MG-63 cells, compared to Ad5-Control-shRNA infected cells. ** *p* < 0.01 (*n =* 3).

To explore the mechanism by which Kv1.3 promotes the growth of osteosarcoma cells, we examined apoptosis following Kv1.3 knockdown by double staining with Annexin V and PI. Ad5-Kv1.3-shRNA infected MG-63 cells demonstrated a significant increase of apoptotic rate compared to Ad5-Control-shRNA infected cells (Figure 4).

### 2.5. Kv1.3 Knockdown Trigger Caspase3/7 Activation

**Figure 5:**
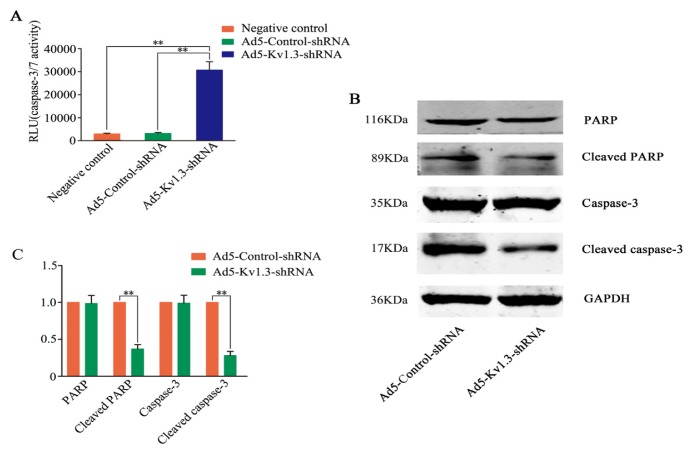
Kv1.3 knockdown leads to the activation of Caspase-3/7 in MG-63 cells. (**A**) The level of activated caspase-3/7 was higher in Ad5-Kv1.3-shRNA infected cells. ** *p* < 0.01, compared with Ad5-Control-shRNA or negative control (NC, cells without infection) (*n* = 3); (**B**) Western blot analysis showed that the levels of cleaved PARP and cleaved caspase-3 were higher in Ad5-Kv1.3-shRNA infected cells than in the control adenoviral vector infected cells, while the levels of PARP and caspase-3 in Ad5-Kv1.3-shRNA and Ad5-Control-shRNA infected cells had no obvious difference; (**C**) Densitometry analysis of the levels of the proteins shown in (**B**), and the results were expressed as mean ± SD (*n =* 3). ** *p* < 0.01. GAPDH was loading control.

The mechanisms of apoptosis are highly complicated and involve two main pathways: the extrinsic pathway and the intrinsic pathway [21]. We next determined the activity of caspase3/7, effector caspases, following Kv1.3 knockdown in MG-63 cells. The amount of activated caspase-3/7 was significantly higher in Ad5-Kv1.3-shRNA infected cells than in Ad5-Control-shRNA infected cells (Figure 5A). Furthermore, we detected PARP cleavage, an indicator of caspase-dependent apoptosis, and found that the level of cleaved PARP was significantly higher in Ad5-Kv1.3-shRNA infected cells than in Ad5-Control-shRNA infected cells. Similarly, the level of cleaved caspase-3 in Ad5-Kv1.3-shRNA infected cells was significantly higher than in Ad5-Control-shRNA infected cells (Figure 5B). These results indicated that knockdown of Kv1.3 by shRNA induces apoptosis of MG-63 cells via the Caspase-3/7 pathway.

## 3. Discussion

Kv channels subtype Kv1.3 has been implicated in the regulation of many cellular functions, including membrane potential, solute and water transport, cell-volume, adhesion, motility, apoptosis and proliferation [22]. Numerous studies have demonstrated that aberrant expression of Kv1.3 is involved in the progression and survival of cancers [10]. However, its function during tumorigenesis is debatable [16,23,24]. Up to now, the expression and function of Kv1.3 in human osteosarcoma remain unknown. Therefore, we investigated the expression and function of Kv1.3 in human osteosarcoma in this study.

By RT-PCR, Western blot, and immunohistochemistry, we found increased expression of Kv1.3 in human osteosarcoma cell line and tissues. Compared with pharmacologic Kv1.3 inhibitors, such as 4-aminopyridine (4-AP) [25], tetraethylammonium (TEA) [25], and margatoxin (MgTX) [26], small interfering RNA (siRNA) is a more specific tool to investigate the role of Kv1.3 in cancer progression, as siRNA mediated knockdown of Kv1.3 resulted in reduced proliferation of tumor cell lines with less nonspecific responses [27]. In our study, Kv1.3-shRNA effectively downregulated Kv1.3 expression and significantly inhibited the growth of osterosarcoma cells *in vitro* and *in vivo*.

Apoptotic cell death is one of the main mechanisms involved in the decrease of cell growth following the inactivation of oncogene or the activation of tumor suppressor gene. To determine whether the growth inhibitory effect of MG-63 cells mediated by Kv1.3-shRNA was related to the induction of apoptosis, we used flow cytometry to examine apoptotic rate in Ad5-Kv1.3-shRNA infected MG-63 cells and found that Kv1.3-shRNA induced a significant increase in apoptosis. The activation of caspases is one of the major processes in apoptosis [28]. Thus, we examined the levels of caspase-3/7, the key apoptotic caspases, and the cleavage of PARP, a marker of apoptosis, in Ad5-Kv1.3-shRNA infected cells. Significant increases of activated caspase-3/7, cleaved PARP and cleaved caspase-3 were observed in Ad5-Kv1.3-shRNA infected cells. These data suggest that inhibition of Kv1.3 leads to the activation of caspase-3/7 and the execution of apoptosis.

Notably, recent data suggest that Kv1.3 is active in the inner mitochondrial membrane. On the induction of apoptosis, Bax on the outer mitochondrial membrane binds to and inhibits Kv1.3, resulting in hyperpolarization, an increase in reactive oxygen species production, and cytochrome c release [13]. Thus, the inhibition of Kv1.3 activity or expression has been proposed as a potential mechanism that could induce apoptosis [14,15]. Consistent with these previous data, in this study we found that knockdown of Kv1.3 induced the apoptosis of osteosarcoma cells.

In conclusion, this is the first study to show that Kv1.3 was upregulated in human osteosarcoma and downregulation of Kv1.3 suppressed osteosarcoma growth *in vivo* and osteosarcoma cell proliferation *in vitro*, accompanied by increased apoptosis. Our findings suggest that Kv1.3 is a novel target for the development of molecular targeted cancer therapies for osterosarcoma.

## 4. Experimental Section

### 4.1. Cell Culture

Human osteosarcoma cell line MG-63, human embryonic kidney cell line 293 (HEK293), human Jurkat cell line TIB-152, and human osteoblastic cell line hFOB 1.19 were obtained from the American type culture of collection (ATCC). MG-63, HEK293, and TIB-152 cells were cultured in RPMI-1640 medium (Gibco, Rockville, MD, USA) supplemented with 10% fetal bovine serum (FBS), 100 U/mL penicillin, and 100 μg/mL streptomycin at 37 °C in a humidified atmosphere with 5% CO2. hFOB 1.19 cells were cultured in ham’sF12/ Dulbecco’s modified Eagle medium (DMEM, Gibco, Rockville, MD, USA) without phenol red supplemented with 10% FBS, 100 U/mL penicillin, and 100 μg/mL streptomycin at 34 °C in a humidified atmosphere with 5% CO_2_. Four cell lines were subcultured every three to four days.

### 4.2. Preparation of Adenoviral shRNA Vectors

The oligonucleotide targeting human Eag was designed and selected as the template: GCA GUG GUA ACC AUG ACA A, which shared no homology with other coding sequences in human genome by BLAST analysis. A ring sequence of nine base pairs (TTC AAG ACG) existed between the sense and antisense strands. The shRNA was synthesized by Sangon Biotech (Shanghai, China). Plasmid pGeneSil-1 containing the human U6 promoter was purchased from GeneSil Biotechnology (Wuhan, China). The shRNA-expressing cassette was subcloned into pAdTrack vector between HindIII and XbaI sites. The recombinant plasmid was linearized by digestion with restriction endonuclease, and subsequently cotransformed into *E. coli* BJ5183 cells with an adenoviral backbone plasmid, pAdEasy-1. Recombinant plasmids were selected for kanamycin resistance, and transduced into HEK293 cells. A recombinant adenovirus expressing shRNA against Kv1.3 (Ad5-Kv1.3-shRNA) was generated. The recombinant adenovirus (Ad5-Control-shRNA), which contained the CTA CCT GTT CTA GTC TGG ACT sequence and did not target any known human genes, was generated as the control for Ad5-Kv1.3-shRNA. All viruses were propagated and purified on a CsCl gradient using standard methods. The viruses were titered for viral particles using standard methods based on spectrophotometry at 260 nm. Functional titer (plaque forming units) was determined with a plaque assay on HEK293 cells according to the method developed by Quantum Biotechnology.

### 4.3. Adenovirus Infection

MG-63 cells (1 × 10^5^) in serum-free RPMI-1640 were infected with Ad5-Kv1.3-shRNA or Ad5-Control-shRNA at 5 MOI (multiplicity of infection, calculated as PFU/cell numbers) in a humidified atmosphere of 5% CO_2_ at 37 °C. Virus-containing medium was removed eight hours later and replaced with fresh RPMI-1640 medium containing 10% (*v/v*) FBS. Cells were incubated for additional 48 h.

The total RNA was isolated from the cultured cells by Trizol reagent (Invitrogen, Rockville, MD, USA). RNA purity and integrity was checked by denaturing 1% (*w/v*) agarose gel. cDNA was synthesized from 1 μg of total RNA using 200 U reverse transcriptase (Takara, Tokyo, Japan) in a 20 μL reaction volume containing 200 μM dNTPs and 2.5 μM oligo-dT primer at 30 °C for 10 min, then at 42 °C for 60 min, and finally at 80 °C for 5 min. One μL of cDNA were amplified by PCR for 35 cycles in 25 μL reaction volume containing 2.5 U DNA polymerase and 200 μM dNTPs. Sequences of forward and reverse primers, amplified fragment sizes, and annealing temperatures were as follows: Kv1.3, 5′-GGT CAT CAA CAT CTCCGG CGT GCG CT-3′, 5′-AGG GCC GCT CCT CCT CCCGC-3′, 67 °C, 314 bp. β-actin, 5′-TCC ACC TTC CAG CAG ATG TG-3′, 5′GCA TTT GCG GTG GAC GAT-3′, 54 °C, 75 bp. PCR products were run on a 2% agarose gel (Sigma, St. Louis, MO, USA) with a 100 bp DNA ladder, and the bands were visualized by ethidium bromide staining on a UV trans illuminator. Each experiment was repeated three times. Some of PCR products were sequenced to check PCR specificity. The relative mRNA level of Kv1.3 was calculated by densitometry analysis of the gels with that of β-actin as the standard.

### 4.4. Western Blot Analysis

About 5–6 × 10^7^ cells were collected and lysed in ice-cold lysis buffer containing 50 mmol/L Tris-Cl (pH 7.5), 150 mmol/L NaCl, 0.2 mmol/L EDTA, 1 mmol/L PMSF and 1% (*v/v*) Nonidet-P40 for 30 min. The lysates were centrifuged at 13,200 rpm for 10 min at 4 °C and the supernatants were collected. 25 μg protein were resolved by a 12% SDS-PAGE and blotted onto nitrocellulose membranes (Bio-Rad, Richmond, CA, USA). Membranes were blocked with 10% (*w/v*) nonfat milk at room temperature for one hour, and then incubated with antibodies against Kv1.3 (Abcam, Cambridge, MA, USA), PARP (Cell Signaling Technology^®^, Danvers, MA, USA), caspase-3, cleaved caspase-3 (Cell Signaling) and glyceraldehyde 3-phosphate dehydrogenase (GAPDH) (Abcam) overnight at 4 °C, followed by incubation with horseradish peroxidase-conjugated goat anti-rabbit or anti-mouse secondary antibody (Santa Cruz Biotechnology, CA, USA) at room temperature for one hour. The membranes were, then, developed with chemiluminescent detection kit (Zhongshan Biotechnology, Beijing, China) and exposed to X-ray films. Experiments were performed at least three times with representative data presented.

### 4.5. Sample Collection and Immunohistochemistry

A total of 41 formalin-fixed, paraffin-embedded osteosarcoma specimens from 22 patients (before the administration of neoadjuvant chemotherapy) were acquired from the Affiliated Southeast Hospital of Xiamen University between January 2009 and June 2012. As a positive control sample [18], confirmed healthy human brain, obtained by biopsy, was used. All the specimens were collected after obtaining written informed consent according to a protocol approved by Institutional Review Board of the Affiliated Southeast Hospital of Xiamen University.

For immunohistochemistry, the tissue slide was baked on a rack in a dry oven at 60 °C for two hours to remove the coated paraffin. The slide was immersed twice in xylene (Zhongshan Biotechnology) for three minutes, hydrated consectively with 100%, 95%, 70%, and 50% ethanol (Zhongshan Biotechnology) and rinsed with cold top water for five minutes. After dewaxing and blocking endogenous peroxidases, the sections were treated at 100 °C in EDTA (1 mM, pH 8.0) for antigen retrieval, and then incubated with anti-Kv1.3 antibody (1:500, Abcam) overnight at 4 °C. The slide was washed with PBS the following day and incubated with biotinylated goat anti-rabbit IgG (Abcam) at room temperature for one hour. Avidin biotin complex (ABC) (Zhongshan Biotechnology) and diaminobenzamidine (DAB) (Zhongshan Biotechnology) were used to visualize the tissue slide according to manufacturer’s instructions, and then the sections were counterstained with haematoxylin.

### 4.6. Cell Proliferation Assay

The cell proliferation was analyzed by using Cell Counting Assay Kit-8 (CCK-8) (Dojindo Molecular Technologies, Gaithersburg, MD, USA) according to the manufacturer’s protocol. In brief, 1 × 10^5^ cells were starved in serum-free medium for 12 h and then the cells were transduced. After 48 h, cells were harvested. Ten microliters of Cell Counting Assay Kit-8 solution was added to each well, the cells were incubated for another hour, and the absorbance (A) at 450 nm was measured by using spectrophotometer (Bio-Rad). Experiments were performed at least three times with representative data presented.

### 4.7. Tumor Xenograft Model

Thymys-null BALB/c nude mice (female, age six to eight weeks) were obtained from the Animal Center of Chinese Academy of Medical Sciences. All animal procedures were performed according to approved protocols and in accordance with recommendations for the proper use and care of laboratory animals. Osteosarcoma xenografts were established in nude mice as previously described [24]. A total of 1.5 × 10^6^ MG-63 cells in 150 μL phosphate-buffered aline (PBS) were subcutaneously injected in the hind right leg. One week later, the tumors grew to visible size. The osteosarcoma-bearing mice were randomly divided into three groups (six in each group). Group 1 received intra-tumor injections with Ad5- Kv1.3-shRNA (10 MOI) every two days (six injections total). Group 2 received intra-tumor injections of Ad5-Control-shRNA (10 MOI) every two days (six injections total). Group 3 received normal saline injection as controls. All treatments were given every other day for a total of seven doses. Tumor volume (cm^3^) was determined based on the following formula: ab^2^/2 where a was the length and b was the width of the tumor [29].

### 4.8. Analysis of Cell Apoptosis

Apoptosis was determined using flpo cytometry and the Apo-ONE homogeneous caspase-3/7 assay (Promega, Madison, WI, USA) according to the manufacturer’s instructions.

For flor cytometry, cells were prepared and analyzed using a FACScan apparatus (Becton-Dickinson, San Jose, CA, USA) following the manufacturer’s protocol. Early apoptotic cells were defined as Annexin V positive and propidium iodide (PI) negative cells.

For caspase-3/7 assay, 20 μL of cell suspension (1 × 10^5^ cells/mL) was seeded into 96-well plates (Sigma, St. Louis, MO, USA) and incubated at 37 °C overnight. After the cells were treated with various adenovirus constructs, an equal volume of Apo-ONE Caspase-3/7-reagent was added to a 96-well plate and incubated at room temperature for one hour. The luminescence of each sample was measured in a plate-reading luminometer (Tecan, Switzerland). Experiments were performed at least three times with representative data presented.

### 4.9. Statistical Analysis

All data were presented as mean ± standard deviation (SD). Statistical significance was determined using *t*-test or analysis of variance (ANOVA) using the SPSS18.0 program. *p* < 0.05 was considered as statistically significant.

## 5. Conclusions

Kv1.3 expression is remodeled during tumorigenesis and is involved in proliferation and apoptosis of human osteosarcoma cells. Thus, Kv1.3 targeting therapy can be a novel strategy for the treatment of osteosarcoma.
